# Extraction and Surfactant Properties of Glyoxylic Acid‐Functionalized Lignin

**DOI:** 10.1002/cssc.202200270

**Published:** 2022-06-07

**Authors:** Stefania Bertella, Monique Bernardes Figueirêdo, Gaia De Angelis, Malcolm Mourez, Claire Bourmaud, Esther Amstad, Jeremy S. Luterbacher

**Affiliations:** ^1^ Laboratory of Sustainable and Catalytic Processing Institute of Chemical Sciences and Engineering École Polytechnique Fédérale de Lausanne (EPFL) CH-1015 Lausanne Switzerland; ^2^ Soft Materials Laboratory Institute of Materials École Polytechnique Fédérale de Lausanne (EPFL) CH-1015 Lausanne Switzerland; ^3^ Department of Chemistry École Polytechnique Institut Polytechnique de Paris 91128 Palaiseau Cedex France

**Keywords:** biomass, functionalization, lignin, surfactants, surface tension.

## Abstract

The amphiphilic chemical structure of native lignin, composed by a hydrophobic aromatic core and hydrophilic hydroxy groups, makes it a promising alternative for the development of bio‐based surface‐active compounds. However, the severe conditions traditionally needed during biomass fractionation make lignin prone to condensation and cause it to lose hydrophilic hydroxy groups in favour of the formation of C−C bonds, ultimately decreasing lignin's abilities to lower surface tension of water/oil mixtures. Therefore, it is often necessary to further functionalize lignin in additional synthetic steps in order to obtain a surfactant with suitable properties. In this work, multifunctional aldehyde‐assisted fractionation with glyoxylic acid (GA) was used to prevent lignin condensation and simultaneously introduce a controlled amount of carboxylic acid on the lignin backbone for its further use as surfactant. After fully characterizing the extracted GA‐lignin, its surface activity was measured in several water/oil systems at different pH values. Then, the stability of water/mineral oil emulsions was evaluated at different pH and over a course of 30 days by traditional photography and microscopy imaging. Further, the use of GA‐lignin as a surfactant was investigated in the formulation of a cosmetic hand cream composed of industrially relevant ingredients. Contrary to industrial lignins such as Kraft lignin, GA‐lignin did not alter the color or smell of the formulation. Finally, the surface activity of GA‐lignin was compared with other lignin‐based and fossil‐based surfactants, showing that GA‐lignin presented similar or better surface‐active properties compared to some of the most commonly used surfactants. The overall results showed that GA‐lignin, a biopolymer that can be made exclusively from renewable carbon, can successfully be extracted in one step from lignocellulosic biomass. This lignin can be used as an effective surfactant without further modification, and as such is a promising candidate for the development of new bio‐based surface‐active products.

## Graphical abstract

**From wood to hand cream**: A method to extract and functionalize lignin in one step from biomass using glyoxylic acid (GA) is reported. The degree of functionalization is controlled, and GA‐lignin can be used directly as a surfactant. The resulting surface‐active properties are explored at different pH for 30 days and compared to bio‐ and fossil‐based surfactants, showing that GA‐lignin is an easily accessible and promising bio‐based surface‐active macromolecule.

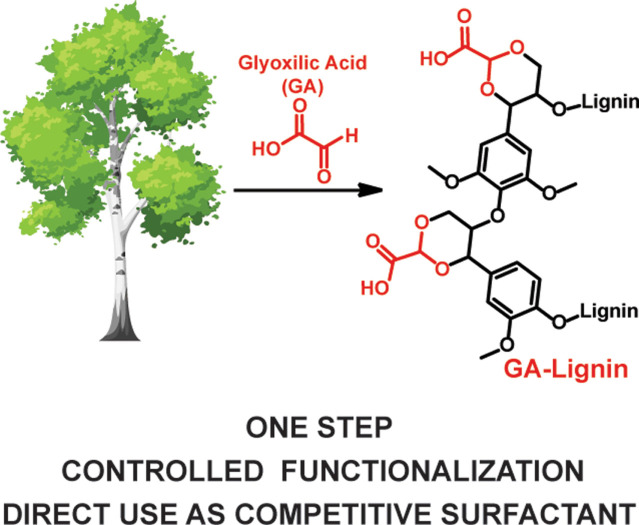

## Introduction

**Figure 1:**
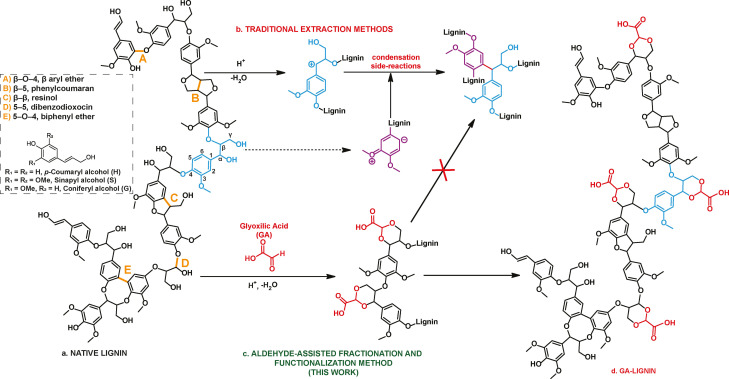
Overview of the lignin extraction and functionalization presented in this work. (a) Structure of native lignin in lignocellulosic biomass. (b) Reactions of condensation and repolymerization in traditional lignin fractionation. (c) Aldehyde‐assisted fractionation with glyoxylic acid. (d) Structure of extracted GA‐lignin that is used as a surfactant.

Lignin is a heterogeneous aromatic biopolymer synthesized in plants from three aromatic C_9_ monolignols: sinapyl, coniferyl, and *p*‐coumaryl alcohols.^[1]^ The ratio of thee monolignols in the final polymer depends on the plant species.^[2]^ For example, softwood like pine is composed only of guaiacyl units, while hardwood like birch contains both guaiacyl and syringyl units.^[3]^ A second source of heterogeneity in lignin comes from its radical biosynthesis, which leads to a polymer where the aromatic units are bound together by an array of different chemical linkages (Figure 1a).^[4]^ Amidst these various linkages, the β‐O‐4 ether bond comprises 50–80 % of woody biomass, and therefore it is frequently targeted for the chemical functionalization of lignin or for the depolymerization of this biopolymer into aromatic monomers.[^5^, ^6^, ^7^] Due to its high natural abundance and unique structure, lignin has therefore been targeted as a potential renewable substitute for fossil‐based aromatic materials.^[8]^ In particular, the aromatic core structure of lignin, coupled with the hydroxy groups along its polymeric chains, makes it a material with a certain degree of amphiphilicity, which could enable its use as a surfactant or dispersant in various fields and applications.[^9^, ^10^, ^11^]

Despite its abundance, lignin extraction from plant material is complex. Traditionally, to separate lignin from cellulose and hemicellulose, the other two main components of lignocellulosic biomass, it is necessary to use high temperatures, between 130–180 °C, and strong pH conditions, such as pH values above 12 for Kraft, and pH ranging from 1 to 5 for the sulfite processes.[^8^, ^12^] While providing the needed energy and catalytic conditions to isolate lignin from the rest of the biomass, these parameters also impede any control over the lignin's final chemical structure. The high temperatures combined with strong acidic or basic pH favor dehydration reactions on the α‐group of the β‐O‐4 linkages, which promote lignin repolymerization and the formation of novel carbon–carbon bonds (Figure 1b).^[13]^ Consequently, the number of hydroxy groups on the biopolymer backbone diminishes, the polymer complexity increases, and the isolated lignin is generally recalcitrant to further valorization. Despite this, some examples of extracted lignins used for the development of bio‐based surfactants have been reported. Soares et al., for example, recently described the use of Kraft lignin extracted from the black liquor of an industrial pulp and paper plant to prepare a sustainable biocide via the stabilization of an aqueous solution of thymol.^[14]^ In addition, lignosulfonates, a by‐product of the sulfite pulping process, have been reported to lower the surface tension of water and oil mixtures, given their aromatic core structure and the presence the sulfonate anionic groups, which are introduced during extraction.[^15^, ^16^]

To increase the properties of lignin as surfactant, chemical modification is consequently often necessary to introduce better hydrophilic groups along the polymer chains.^[17]^ For instance, reactions of sulfethylation,^[18]^ esterification,^[19]^ etherification,^[20]^ as well as grafting through reversible addition‐fragmentation chain‐transfer (RAFT) polymerization^[21]^ have been carried out on lignin oligomers to explore their further use as surfactants.

Examples of these approaches relying on post‐extraction chemical functionalization of lignin have been reported to produce pH‐dependent emulsions with tunable physical properties^[22]^ as well as other lignin‐based surfactants with UV‐shielding properties,^[23]^ which could be then employed to develop cream‐based formulations.[^24^, ^25^] While most of these approaches have delivered good results in terms of surface‐active properties, stability, and UV‐shielding factors, the same properties were only achieved after performing several synthetic steps on the extracted lignin. These steps were often performed with fossil‐based and/or toxic reagents, which likely decreases any environmental benefit of using the final products.^[26]^

Directly functionalizing lignin during its extraction could avoid its condensation and also be used to directly impart the needed functionality to form stable emulsions, which would avoid the need for additional functionalization steps.

We have recently shown that a great degree of control over the extracted lignin chemical structure could be obtained by introducing aldehydes during the biomass fractionation process.[^27^, ^28^] This process, defined as aldehyde‐assisted fractionation (AAF), avoids the condensation and repolymerization reactions on the lignin scaffold by the formation of stable acetals between the β‐O‐4 linkages and the aldehydes introduced during the biomass fractionation (Figure 1c).^[29]^ Moreover, when multifunctional aldehydes are used, it is consequently possible to introduce chemical functionalities on the lignin backbone that were not present in its original structure, tuning therefore the final properties of the isolated material.[^30^, ^31^]

Here, we take advantage of the AAF process to extract lignin in presence of glyoxylic acid (GA), in order to produce a lignin (GA‐lignin) containing a controlled amount of carboxylic acids on its polymeric chains, in a single step from lignocellulosic biomass (Figure 1d). This process allowed us to avoid performing post‐isolation functionalization reactions, which are typically necessary when technical lignins are used in material applications.

We then investigated the surface activity properties of GA‐lignin in several water/oil systems and evaluated the stability of water/mineral oil emulsion using GA‐lignin as a surfactant at different pH values over time. After this, we used GA‐lignin as an active component in the preparation of a cosmetic formulation of hand‐cream. Finally, we benchmarked the surface activity of GA‐lignin against other lignin‐based as well as non‐bio‐based industrial surfactants.

## Results and Discussion

### Extraction and characterization of glyoxylic acid‐functionalized lignin

We extracted GA‐lignin from birch wood (*Betula Pendula*), following a previously published procedure for AAF with some modifications^[32]^ (see detailed procedure in Experimental Section). Briefly, 5 g of wood chips, glyoxylic acid monohydrate, 0.8 mL of HCl 37 wt % in water (acting as the acid catalyst), and 25 mL dioxane were inserted in a reagent bottle equipped with a cap and a magnetic stirrer. The reaction was run at 85 °C for 3 h under vigorous stirring to both favor the full fractionation of the biomass components and to maximize the formation of acetals between glyoxylic acid and the lignin. After filtration of the reaction mixture, the cellulose‐rich fraction was isolated as a solid via filtration. The organic filtrate was first concentrated and then precipitated in 850 mL distilled water to obtain the insoluble GA‐lignin as a fine powder, which was then filtered and dried at 45 °C under vacuum before characterization.

**Figure 2:**
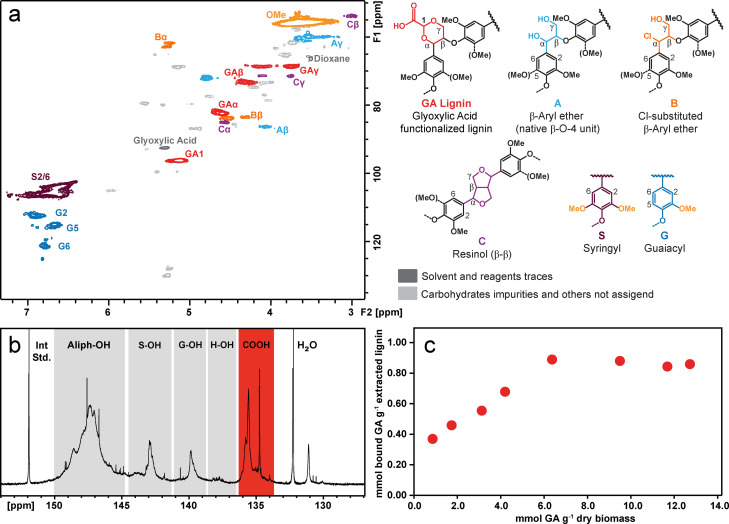
Characterization of GA‐lignin. (a) HSQC NMR with peaks assignment. (b) Example of a ^31^P NMR spectrum with area assigned to the P‐functionalized carboxylic acids signal highlighted in red. (c) Effect of initial GA concentration over the final GA‐functionalization of the extracted lignin measured by ^31^P NMR spectroscopy.

We characterized the resulting lignin via heteronuclear single quantum coherence (HSQC) nuclear magnetic resonance (NMR) spectroscopy (Figure 2a), for which it was possible to assign all the signals relative to the different linkages present in the hardwood lignin after extraction. The extracted GA‐lignin spectrum presented all the characteristic signals of AAF‐functionalized lignin, as well as some residual carbohydrate impurities and GA (see Supporting Information 2.4.3). In the NMR spectrum, the peak corresponding to the newly formed acetals at *δ*_H_/*δ*_C_=4.86/96.2 ppm confirmed that glyoxylic acid had successfully reacted with the lignin. However, the HSQC spectrum also contained evidence that the aldehyde incorporation was incomplete, as the signals corresponding to the native β‐O‐4 bonds were still visible. In addition, the signals at *δ*_H_/*δ*_C_=4.92/62.1 and 4.08/83.5 ppm were attributed to the incorporation of a chlorine atom in the α‐position of the β‐O‐4 bond, in a competing mechanism to the acetalization reaction, as also reported by Zijlstra et al.^[33]^ This side reaction has generally not been previously observed as one of the main side‐reactions when lignin was extracted in presence of other aldehydes such as formaldehyde or propionaldehyde,^[28]^ suggesting that GA was slightly less effective than other aldehydes at forming acetals with the lignin polymer. However, as traditional HSQC NMR experiments only provide qualitative structural information, we decided to quantify the amount of GA present on the lignin by using the ^31^P NMR protocol for lignin hydroxy groups determination developed by Meng et al.^[34]^ From the ^31^P NMR spectrum (Figure 2b), we could identify the signals relative to the aliphatic groups as well as that of the free phenols of the syringyl, guaiacyl, and *p*‐hydroxyphenyl units. As expected, the ^31^P NMR spectrum of GA‐lignin also presented strong signals centered at 135 ppm relative to the newly introduced carboxylic acid groups (highlighted in red on Figure 2b) that could be quantified by using a known amount of *N*‐hydroxy‐5‐norbornene‐2,3‐dicarboximide (NHND) as an internal standard. We then decided to verify if we could control the final chemical functionalization of the lignin by varying the amount of GA introduced in the reactor during the biomass fractionation, as we had previously shown was possible with terephthalic aldehyde (TALD).^[30]^ Such control also proved to be successful for GA (Figure 2c). The correlation between the amount of GA used during the fractionation reaction versus the mmol of carboxylic acids quantified on the extracted lignin by ^31^P NMR was close to linear before reaching a plateau at around 6.5 mmol of GA g^−1^ of dry biomass, similarly to what we observed in the case of TALD‐lignin,^[30]^ confirming the potential of controlling the chemical functionalities of lignin during the extraction process.

### Surface tension measurements

**Figure 3:**
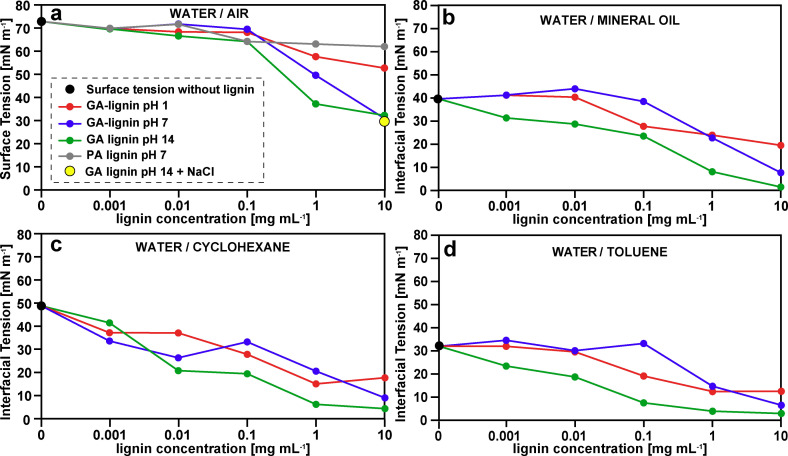
Concentration‐dependent surface tension measurements of water containing GA‐lignin at different pH values (pH 1 red lines, pH 7 blue lines, pH 14 green lines) and different oil phases: (a) air, (b) mineral oil, (c) cyclohexane, and (d) toluene. Black dots correspond to the initial interfacial tension values (without lignin), the grey line corresponds to PA‐lignin, and the yellow dot corresponds to a GA‐lignin sample at pH 14 with additional NaCl.

Given the hydrophobic aromatic backbone of GA‐lignin and the newly introduced hydrophilic carboxylates through AAF, we exploited these features for use of GA‐lignin as a new bio‐based surfactant. Specifically, we measured if and how the presence of GA‐lignin, extracted with 14 mmol GA g^−1^ dry biomass, could lower the surface/interfacial tension of different water/air or water/oil systems in acid, basic, or neutral pH, compared to the same systems without the presence of lignin. GA‐lignin showed the ability to lower the interfacial tension of all these systems regardless of the pH at which the measurements were taken (Figure 3). In particular, we observed that for the water/air system GA‐lignin could lower the surface tension up to 57 % at a concentration of 10 mg mL^−1^ (Figure 3a). At the same concentrations, GA‐lignin lowered the interfacial tension of water/cyclohexane (Figure 3c) and water/toluene systems (Figure 3d) up to 92 and 91 %, respectively. Finally, in the water/mineral oil system (Figure 3b), the presence of GA‐lignin was able to lower the surface tension up to 96 %, from the starting value of 40 mN m^−1^ to the final value of 2 mN m^−1^.

Generally, we observed that a higher solution pH led to lower values of interfacial tension. This could be explained by the different degree of protonation that the chemical features of GA‐lignin present at pH 14, 7, and 1. In particular, at pH 14 both carboxylates and phenolics are in a deprotonated form, at pH 7 only the carboxylates are deprotonated, while at pH 1 both phenolics and carboxylic acid are fully protonated. Therefore, at high values of pH the amphiphilic characteristics of fully deprotonated GA‐lignin are enhanced compared to its protonated form in a strong acidic environment.

To verify the hypothesis that carboxylates had an active role in lowering the values of interfacial tension, we also performed a control experiment by repeating the same measurements, but using instead a lignin extracted in presence of propionaldehyde (PA‐lignin), which did not have any added carboxylate functionalities (Figure S1). The addition of PA‐lignin to the water system at pH 7 had a minimal effect on the values of measured interfacial tension, which was only lowered by 15 % at 10 mg mL^−1^ (Figure 3a, grey line), confirming the hypothesis that carboxylate groups are pivotal to provide a system with good amphiphilic properties.

As the sample preparation (see Experimental Section) involved a first complete dissolution of the GA‐lignin in 1 m NaOH, followed by adjustment with 0.1 m HCl to reach the desired pH values, NaCl was generated. The presence of the additional salt could potentially influence the measured interfacial tension values. Therefore, we performed an additional control measurement on a sample that was prepared by first dissolving GA‐lignin in 1 m NaOH at pH 14, and then by adding the same amount of NaCl that a sample prepared with pH adjustment to 7 would contain. From the surface tension measurements (Figure 3a, yellow dot), we did not observe any strong salt influence on the value of interfacial tension, as the difference between samples at pH 14 with or without NaCl was only 3 mN m^−1^.

We also measured the values of CMC (critical micelle concentration) for GA‐lignin at pH 14, at which point the high deprotonation makes GA‐lignin soluble in aqueous solutions. The presence of fully soluble lignin ensured that no Pickering emulsions were present at the same time as micelles in solution, which would complicate the measurement of a CMC. The values of surface tension appeared to reach a plateau for lignin concentration equal to or higher than 1 mg mL^−1^ (0.1 wt %), indicating that the CMC is likely reached at this concentration (Figure 3).

We compared these measured values with the CMC of other synthesized lignin‐based surfactants recently reported in literature. In particular, Delgado et al. observed that the values of CMC for Kraft lignin modified with succinic anhydride or dodecyl succinic anhydride presented a CMC at around 0.2 wt % at a surface tension between 35–40 mN m^−1^.^[35]^ Similar values of surface tension were measured by Zhang. et al. for alkaline lignin modified through amination, sulfonation, and acylation, but with a CMC of 2.5 wt %.^[17]^ Finally, Chen et al. described the synthesis of lignin polyether sulfonates that had a surface tension comprised between 33 mN m^−1^ with a CMC of 0.5 wt %.^[36]^ These results indicated that GA‐lignin, with a surface tension of 32 mN m^−1^ at a CMC of 0.1 wt %, performed as well as or better than other lignin‐based reported surfactants.

That being said, GA‐lignin offers the unique advantage that no additional synthetic steps are needed after its isolation to impart good surface‐active properties to the resulting material, making the process of surfactant production significantly simpler compared to the other reported lignin‐based surfactants.

### Microscopy imaging of water/mineral oil emulsions over time

We investigated the stability of the emulsions formed by adding 1 mL of water containing GA‐lignin at a concentration of 10 mg mL^−1^ and at different pH values (1, 7, 14) or without lignin at pH 7 for the control experiment to 0.5 mL of mineral oil, which is a common ingredient in the preparation of creams and lotions for skin care and cosmetic applications.

Since the surfactant was in the continuous aqueous phase, which was in excess compared to the oil phase, the produced emulsions were expected to be oil in water. This hypothesis was confirmed by fluorescence microscopy, where it was possible to see that the fluorescent lignin was only visible in the continuous aqueous phase and not in the organic inner phase (Figure S5).

**Figure 4:**
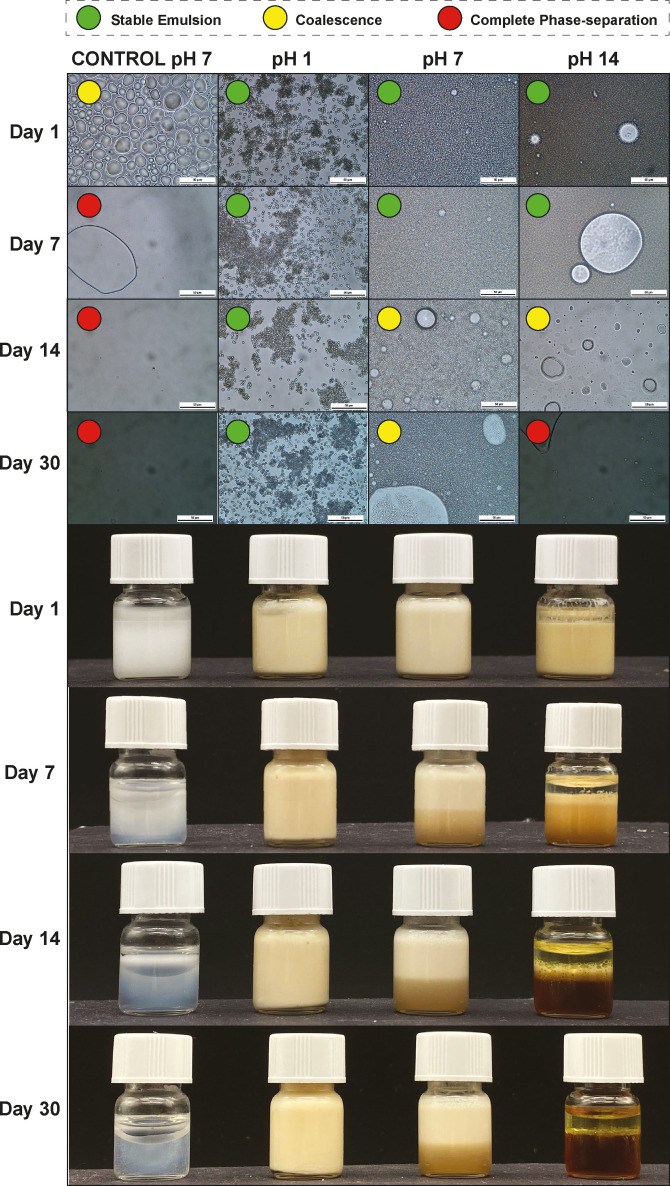
Images of different water/mineral oil emulsions at different pH taken over the course of 30 days: (top) taken with optical microscopy with a scale bar of 50 μm and colored dots to mark the stability of the emulsions; and (bottom) taken with traditional photography of the full vials containing the emulsions.

We also monitored the emulsions over the course of 30 days by both inspection with the naked eye and with optical microscopy (Figure 4). Unsurprisingly, the control water/mineral oil emulsion that did not contain any GA‐lignin had very poor stability and started to coalesce shortly after preparation, with complete phase separation already at day 7. The emulsions that contained GA‐lignin as the surfactant had instead a higher stability, although this stability decreased with increasing values of pH. The system prepared at pH 14 formed emulsions that were stable for two weeks and started to coalesce thereafter, reaching full phase separation at day 30. This could be explained by the fact that at high pH the GA‐lignin is fully deprotonated and therefore the different lignin oligomers electrostatically repel each other, preventing the molecules from tightly packing at the interface, which could decrease emulsion stability. The estimated diffusion coefficient (*D*) of GA‐lignin at pH 14, calculated via diffusion‐ordered spectroscopy (DOSY) NMR measurements increased over 14 days, suggesting that the instability of the emulsions could also be due to partial depolymerization of the lignin oligomers at these high pH values (see Supporting Information 2.4.2).

Work by Lancefield et al. explored the fractionation of Kraft lignin and the determination of the diffusion coefficient *D* of each fraction by DOSY NMR spectroscopy, as well as the simultaneous measurement of number‐ and weight‐average molecular weight (*M*_n_ and *M*_w_) by gel‐permeation chromatography (GPC).^[37]^ To estimate the differences in molecular weight in our system, we compared Kraft lignin fractions having the same values of *D* as we measured for GA‐lignin at pH 14 at day 1 and 14, and we assumed that the difference in *M*_n_ and *M*_w_ would be similar. Even though this approach has some limitations, especially for the different type of lignin and solvent systems, we can use this approach to estimate that the change in diffusion coefficient *D* observed here likely corresponds to a decrease in *M*_n_ and *M*_w_ of 10–15 %, which is relatively small considering that lignin extracted through AAF has approximately an initial *M*_n_ of 2500 g mol^−1^ and an *M*_w_ of 6100 g mol^−1^.^[30]^

The emulsions at pH 7 and 1 both showed higher stability. Even though some coalescence was visible for the system prepared at pH 7, the presence of emulsions was still confirmed at day 30 with the optical microscope images. At pH 1, the system appeared completely stable over the course of the 30 days, and the photographs of the prepared emulsions in the vial as well as the microscopy images did not change in appearance. However, from the microscope images, we clearly observed that this system was behaving differently, as some aggregates could be observed. We propose that at pH 1 GA‐lignin was not only in the aqueous solution but started also to form a colloid of aggregates, which generated a Pickering emulsion as previously reported in literature.[^22^, ^38^] We confirmed the presence of aggregates by performing dynamic light scattering (DLS) experiments on the aqueous phases at different pH values containing GA‐lignin (see Supporting Information S2.5). Even though DLS does not give information about their concentration, the sample at pH 1 showed the presence of bigger particles compared to the solutions at lower pH, which was consistent with the formation of Pickering emulsions and the different behavior and higher stability of emulsion prepared at low pH. In conclusion, the systems at neutral and acidic pH showed high stability, most likely due to the formation of Pickering emulsions. This high stability (which was confirmed by an extra set of photos and microscopy images taken at 180 days and shown in Figure S6) opens up the possibility of using this type of lignin for cosmetic application, for example, in the preparation of creams and lotions.

### Preparation of a hand‐cream formulation using GA‐lignin as surfactant

**Figure 5:**
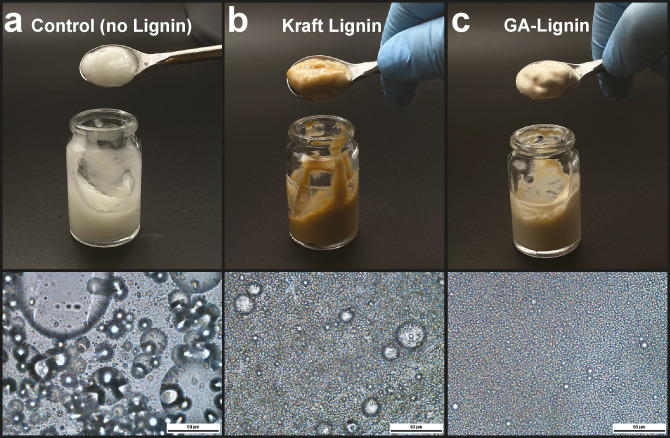
Photographs (top) and microscopy images with a scale bar of 50 μm (bottom) of creams prepared by using water, mineral oil, lignin, xanthan gum, and citral. Sample (a) is a control cream prepared without lignin, sample (b) contains Kraft lignin as surfactant, and sample (c) contains GA‐lignin as surfactant.

Based on the results obtained on the surfactant stability at neutral and acidic pH, we then explored the use of GA‐lignin as surfactant in the preparation of cosmetics. Specifically, we simulated the preparation of a simple hand‐cream made of ingredients commonly used in the cosmetic industry (Figure 5) by mixing water at pH 4 containing 1 wt % of GA‐lignin as the surfactant, and mineral oil as the moisturizer containing 1 wt % of citral (3,7‐dimethyl‐2,6‐octadienal) as a scent. The ratio of the mixed water and oil phases was 2 : 1. Finally, xanthan gum was added as thickener. For this experiment we also prepared two controls: one that had the same composition but did not contain any lignin (Figure 5a), and one where the GA‐lignin was substituted by industrial Kraft Lignin (Figure 5b).

From the microscopy images taken just after the cream preparation, we noticed that the emulsions formed in the control experiment without any surfactant formed bigger and heterogeneous droplets (Figure 5a), confirming again the need for a surfactant to make a homogeneous and stable preparation. The creams that contained Kraft or GA‐lignins had a better texture and generally formed more homogeneous emulsions. However, we could easily observe how the control cream containing industrial Kraft lignin (Figure 5b) had an altered color from the presence of the dark lignin. Moreover, sample B had the very distinctive smell of Kraft lignin, which is usually associated with smoked wood and sulfur containing compounds, which completely covered the smell of citral. In contrast, the hand cream preparation made with GA‐lignin (Figure 5c) yielded a cream where the color was minimally altered and the citral scent was not noticeably altered by the presence of lignin. As a further study on the effect of lignin on the color of the cream, we measured the lignin's UV/Vis absorbance in the range of 200–900 nm in water solutions at pH 4 (see Supporting Information S2.8) and observed that Kraft lignin had a slightly higher absorbance in the visible range than GA‐lignin (at equal concentrations), in accordance with the darker color observed in the creams (Figure S7). Moreover, the high absorbance in the UV range suggested that both lignins (when in the creams) likely lead to excellent UV shielding properties. We finally studied the stability of the GA‐lignin cream over time, observing how this emulsion was extremely robust and stable (no change in appearance for over 6 months).

### Surface tension comparison of GA‐lignin with other lignin‐based and industrial surfactants

**Figure 6:**
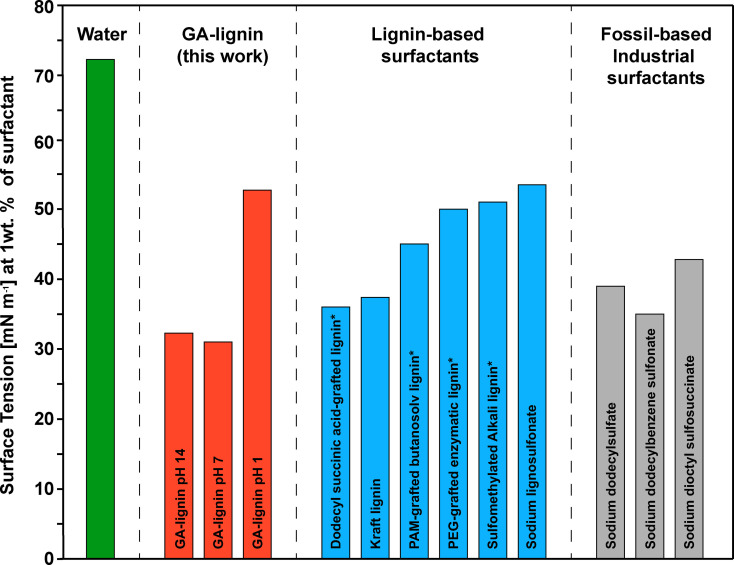
Comparison of the water/air surface tension using various surfactants. Green bars: water/air surface tension without added surfactants. Red bars: water/air surface tension with GA‐lignin at different pH values. Light‐blue bars: water/air surface tension with industrial or chemically modified lignin. Grey bars: water/air surface tension with industrial fossil‐based surfactants. All measurements were done at a surfactant concentration of 10 mg mL^−1^ and at pH 7 if not indicated otherwise. An asterisk (*) indicates that the values were taken from literature. Dodecyl succinic acid‐grafted lignin: Ref. [35]; PAM‐grafted butanosolv lignin: Ref. [40]; PEG‐grafted enzymatic lignin: Ref. [23]; sulfomethylated alkali lignin: Ref. [39].

After demonstrating the ability of GA‐lignin to lower the water/air surface tension and to be a valuable ingredient in the formation of stable formulations of cosmetics, we decided to benchmark the ability of GA‐lignin to lower the surface tension of the water/air system compared to other lignin‐based surfactants commercially available or previously reported in literature, and to common non‐biobased industrial surfactants. To do so, we reproduced and characterized certain lignin‐based surfactants using our same setup and experimental conditions to ensure that any difference was not an artifact of the measurements (Figure 6 and Table S5). As previously shown, GA‐lignin (Figure 3a and Figure 6, red bars, and Table S5) was able to lower the surface tension of the water/air from 72.8 (Figure 6, green bar) to 31 mN m^−1^ regardless of the pH of the aqueous phase. Specifically, GA‐lignin at pH 7 and 14 lowered the surface tension as well as or more than all the other lignins, such as the industrial Kraft lignin and lignosulfonates, or literature‐reported lignins that underwent further chemical modification to impart them with surfactant properties including sulfomethylated lignin,^[39]^ dodecyl succinic acid‐,^[35]^ polyethylene glycol (PEG)‐,^[23]^ or polyacrylamide (PAM)‐^[40]^ grafted lignins (Figure 6, blue bars).

Average GA‐lignin at pH 7 and 14 also led to similar values of surface tension when compared to widely used non‐biobased industrial surfactants at the concentrations of 10 mg mL^−1^, such as sodium dodecylsulfate (SDS), sodium dodecylbenzene sulfonate (SDBS), and sodium dioctyl sulfosuccinate (DOSS) (Figure 6, grey bars). Although GA‐lignin at pH 1 appears to generally perform poorly, the measured value of surface tension of the water/air system is not entirely indicative of its real performance. As previously discussed, at low values of pH the emulsification mechanism of GA‐lignin is different than at higher pHs, with the formation of aggregates and therefore of Pickering emulsions (Figure 4), the stability or formation of which is only partly correlated to the interfacial tension.^[41]^

Overall, the advantages of GA‐lignin compared to other options come from the fact that in order to extract this type of material it is not necessary to use sulfur‐containing molecules, as in the case of Kraft or lignosulfonates, which limits the associated drawbacks related to smell.^[42]^ Moreover, GA‐lignin is directly functionalized during its extraction, which avoids the need to perform further time‐ and energy‐consuming chemical modifications to enhance its properties. Finally, in comparison to fossil‐based surfactants, GA‐lignin could be entirely bio‐sourced, as glyoxylic acid can be produced by reduction of CO_2_‐derived oxalic acid^[43]^ or from the oxidation of bio‐based ethylene glycol.^[44]^ Even though biocompatibility and overall economics of GA‐lignin are still under evaluation,^[45]^ the data shown here confirm how this type of material could facilitate the transition from fossil to bio‐based materials.

## Conclusions

In this work we showed that glyoxylic acid‐functionalized (GA‐)lignin could be extracted in one step from lignocellulosic biomass and that the final degree of functionalization could be tuned and controlled during the fractionation process. GA‐lignin was successfully used as a surfactant, showing an ability to lower the water/air surface tension that was similar or better compared to other chemically modified lignins and fossil‐based surfactants. Imaging of emulsions with mineral oil, cyclohexane, or toluene showed that these emulsions could be stable for more than 30 days, and that the pH had an influence on the formation of traditional or Pickering emulsion. GA‐lignin was then used in the preparation of a simple hand‐cream by mixing ingredients widely exploited in the cosmetic industry. The resulting hand cream formed a stable emulsion where, in contrast to the use of sulfur‐containing Kraft lignin, both color and smell were not affected by the presence of GA‐lignin.

These lignins have the added benefit that they can be produced in concert with highly digestible cellulose and GA‐stabilized xylose, which has been directly used for the production of sustainable bioplastics.^[45]^ Overall, the chemistry shown in this work could allow both the tailoring of lignin properties and the straightforward valorization of all major biomass fractions, which could ultimately make biorefineries more profitable and sustainable.

## Experimental Section

### GA‐lignin extraction

Approximately 5 g of birch wood chips (6.09 % hydration), glyoxylic acid monohydrate (see ratios in Table S1), 0.8 mL of HCl 37 wt % in water, and 25 mL of dioxane were introduced in a 100 mL flat‐bottomed flask along with a 30 mm long magnetic stirrer. The cap was firmly screwed, and the flask was heated at 85 °C in an oil bath. The stirring was gradually increased from an initial rate of 500 to 700 rpm over the course of 1 h. After 3 h of reaction, the flask was removed from the oil bath and allowed to cool to room temperature. The reaction mixture was then filtered with filter paper (Macherey‐Nagel, MN640d) to remove the cellulose‐rich solids. The retentate was washed three times with dioxane to remove all soluble compounds. The filtrate, containing the lignin, hemicellulose, and residual unreacted GA, was then concentrated on a rotary evaporator at 45 °C and a pressure of 90 mbar. For the isolation of GA‐lignin, a 1 L flat‐bottomed flask was filled with approximately 850 mL of deionized water. The water was stirred with a 50 mm bar stirrer at 400 rpm, and the concentrated solution was introduced dropwise, causing the lignin to precipitate as a fine powder. The water solution was then left to stir for 45 min. Finally, the solution was filtered on a 0.8 μm Nylon filter to separate the GA‐lignin from the water‐soluble hemicellulose fraction. The lignin was dried for at least 24 h in a vacuum oven at 45 °C before use.

### HSQC NMR spectroscopy

The NMR samples were made by dissolving 50 mg of lignin in 0.5 mL of deuterated DMSO‐d_6_. A Bruker AV‐ Neo (AV‐III) spectrometer equipped with a 5 mm BBO probe‐head capable of producing magnetic field pulse gradients in the *z*‐direction of 54 G cm^−1^ was used to record all NMR spectra. Frequencies were 400.03 MHz for ^1^H NMR and 100.58 MHz for ^13^C NMR. The HSQC spectrum of the GA‐lignin was also recorded using the standard pulse sequences from Bruker, except that 32 scans were used. All the spectra were then processed using the software Bruker TopSpin 3.6.1.

### Quantitative ^31^P NMR spectroscopy

Quantitative ^31^P NMR spectroscopy was performed following a procedure published by Meng et al. working in a nitrogen atmosphere.^[46]^ Briefly, after drying the lignin samples overnight at 45 °C in a vacuum oven, approximately 30 mg of lignin were inserted in a glass vial equipped with a magnetic stir bar and closed with a polytetrafluoroethylene (PTFE) septum sealed cap. To this vial, we added 0.1 mL of a solution of deuterated pyridine and CDCl_3_ (1.6 : 1, *v*/*v*) containing chromium(III) acetylacetonate solution (≈5.0 mg mL^−1^,) and NHND as an internal standard (≈18.0 mg mL^−1^) with a gas‐tight syringe. To the vial were then added 0.5 mL of a solution of deuterated pyridine and CDCl_3_ (1.6 : 1, *v*/*v*), and the lignin was allowed to fully solubilize while stirring. After complete solubilization of the lignin, 0.1 mL of 2‐chloro‐4,4,5,5‐tetramethyl‐1,3‐2‐dioxaphospholane (TMDP) was added dropwise to the solution. The mixture was stirred for an additional hour to achieve complete derivatization and then transferred to an NMR tube previously dried at 120 °C and equipped with a rubber septum. The samples were analyzed within 3 h of their preparation. The ^31^P NMR spectra were recorded on a Bruker Avance 600 MHz spectrometer equipped with a 5 mm BBO cryoprobe. The experimental parameters used for the spectra acquisition were: pulse program=inverse gated decoupling pulse (zgig), SW=100 ppm, O1P=140 ppm, AQ=0.8 s, D1=10 s, NS=128.

### Sample preparation for surface and interfacial tension measurements

GA‐lignin solutions at five different concentrations (10, 1, 0.1, 0.01, and 0.001 mg mL^−1^) and three different pHs (1, 7, and 14) were prepared in the following manner. First, a starting solution of 20 mg mL^−1^ of GA‐lignin at pH 14 was prepared by dissolving 200 mg of lignin in a 10 mL volumetric flask using a 1 m NaOH solution. Then, using an autopipette (VWR SignatureTM Ergonomic High‐Performance Single‐Channel Variable Volume Pipettor, 10 mL), 2 mL of solution were transferred to a first vial (Vial 1) and 3 mL of solution to a second vial (Vial 2). The remaining 5 mL were left in the original vial, labelled Vial 3. To create the five GA‐lignin solutions at pH 14, 5 mL of a 1 m NaOH solution were first added to Vial 3 using an autopipette. This generated the 10 mg mL^−1^ GA‐lignin solution, which was then successively diluted with the 1 m NaOH solution 4 times using a 10 mL volumetric flask in order to create the 1, 0.1, 0.01, and 0.001 mg mL^−1^ solutions of GA‐lignin at pH 14. To create the five GA‐lignin solutions at pH 7, the pH in Vial 2 was brought down to 7 using HCl, and then the total volume of the solution was adjusted to 6 mL with deionized water. This 10 mg mL^−1^ GA‐lignin solution was then successively diluted with deionized water 4 times using a 10 mL volumetric flask in order to create the 1, 0.1, 0.01, and 0.001 mg mL^−1^ solutions of GA‐lignin at pH 7. To create the five GA‐lignin solutions at pH 1, the pH in Vial 1 was brought down to 1 using HCl, and then the total volume of the solution was adjusted to 4 mL with a 0.1 m HCl solution. This 10 mg mL^−1^ GA‐lignin solution was then successively diluted with the 0.1 m HCl solution 4 times using a 10 mL volumetric flask in order to create the 1, 0.1, 0.01, and 0.001 mg mL^−1^ solutions of GA‐lignin at pH 1. In addition to these samples, a control was prepared by using 1 mL of deionized water at pH 7 without lignin. A second control sample was prepared with GA‐lignin at pH 14 with added NaCl (22.4 mg NaCl mL^−1^ of solution) to match the exact amount of salt formed when adjusting the pH from 14 to 7 with HCl 0.1 m.

### Surface tension measurements

The water/air surface tension or water/oil interfacial tension of each GA‐lignin solution were measured using the pendant drop method. A sample of each solution was inserted in a 1 mL syringe and loaded onto a Krüss DSA 30 drop shape analyzer. The drops were created and visualized using the Krüss Advance software (v.1.6.2.0). Each surface/interfacial tension value was obtained using a standardized procedure. First, a drop was created and expanded until it fell. The volume at which the drop fell was recorded. Then, a second drop was created that had approximately 90 % of the volume of the first drop as measured by the Krüss software. The drop was left to stabilize for three minutes before measuring the surface or interfacial tension value.

### Emulsification tests

1 mL of a 10 mg mL^−1^ GA‐lignin aqueous solution at pH 14, 7, or 1 was added to 0.5 mL of oil phase in a 2 mL glass vial. The GA‐lignin solutions at different pH were made by first preparing a 20 mg mL^−1^ solution at pH 14, and then either diluting it with 1 m NaOH (for the pH 14 solution) or adjusting the pH to the requisite value with HCl and diluting the resulting solution with deionized water (for the pH 7 solution) or 0.1 m HCl (for the pH 1 solution).

The water and oil phases were then tip sonicated with a Branson 450 Digital Sonifier, using the following parameters: 15 s total sonication time, amplitude of 20 %, 2 s on/5 s off pulse sequence.

### Microscopy imaging of emulsions and photos of emulsion vials over time

A Nikon Eclipse TS100 Inverted Microscope was used to view the previously described emulsions. Pictures were taken immediately after the emulsification and every seven days thereafter in order to observe the evolution of the droplets and gauge the stability of the emulsions. Photos of the vials containing the emulsions were taken with an iPhone 11 Pro on the same days as the microscopy images.

### Preparation of a hand‐cream formulation

The hand‐cream formulation was prepared by introducing 9 mL of an aqueous solution of GA‐lignin at a concentration of 1 wt % at pH 4 into a 20 mL glass vial. To this were added 4.5 mL of mineral oil containing 1 wt % of citral and 100 mg of xantham gum. The mixture was then tip sonicated with a Branson 450 Digital Sonifier, using the following parameters: 15 s total sonication time, amplitude of 20 %, 2 s on/5 s off pulse sequence. Two control experiments were also prepared, one without lignin and one by substituting GA‐lignin with Kraft lignin at the same concentration.

## Conflict of interest

S.B. and J.S.L. are inventors on a European patent application (EP19202957) that was submitted by EPFL and covers the isolation of different functionalized lignins via the aldehyde‐assisted process. J.S.L. is co‐founder and part owner of Bloom Biorenewables Ltd that aims at commercializing the aldehyde‐assisted fractionation process.

1

## Supporting information

As a service to our authors and readers, this journal provides supporting information supplied by the authors. Such materials are peer reviewed and may be re‐organized for online delivery, but are not copy‐edited or typeset. Technical support issues arising from supporting information (other than missing files) should be addressed to the authors.

Supporting Information

## Data Availability

The data that support the findings of this study are available on request from the corresponding author. The data are not publicly available due to privacy or ethical restrictions.

## References

[1] Q. Liu , L. Luo , L. Zheng , Int. J. Mol. Sci. 2018, 19, 335. 10.3390/ijms19020335 PMC5855557 29364145

[2] R. Whetten , R. Sederoff , Plant Cell 1995, 7, 1001–1013. 12242395 10.1105/tpc.7.7.1001 PMC160901

[3] J. R. Obst , Holzforschung 1982, 36, 143–152.

[4] W. Boerjan , J. Ralph , M. Baucher , Annu. Rev. Plant Biol. 2003, 54, 519–546. 14503002 10.1146/annurev.arplant.54.031902.134938

[5] M. T. Amiri , S. Bertella , Y. M. Questell-Santiago , J. S. Luterbacher , Chem. Sci. 2019, 10, 8135–8142. 31857880 10.1039/c9sc02088h PMC6836972

[6] W. Lan , Y. Peng Du , S. Sun , J. B. de Bueren , F. Héroguel , J. S. Luterbacher , Green Chem. 2021, 23, 320–327.

[7] T. Renders , G. Van den Bossche , T. Vangeel , K. Van Aelst , B. Sels , Curr. Opin. Biotechnol. 2019, 56, 193–201. 30677700 10.1016/j.copbio.2018.12.005

[8] S. Bertella , J. S. Luterbacher , TRECHEM 2020, 2, 440–453.

[9] N. Alwadani , P. Fatehi , Carbon Resour. Convers. 2018, 1, 126–138.

[10] S. A. Gundersen , J. Sjöblom , Colloid Polym. Sci. 1999, 277, 462–468.

[11] C. Xu , F. Ferdosian , in Conversion of Lignin into Bio-Based Chemicals and Materials (Eds.: C. Xu , F. Ferdosian ), Springer, Berlin, Heidelberg, 2017, pp. 81–90.

[12] D. R. Lobato-Peralta , E. Duque-Brito , H. I. Villafán-Vidales , A. Longoria , P. J. Sebastian , A. K. Cuentas-Gallegos , C. A. Arancibia-Bulnes , P. U. Okoye , J. Cleaner Prod. 2021, 293, 126123.

[13] L. Shuai , M. Talebi Amiri , J. S. Luterbacher , Curr. Opin. Green Sustain. Chem. 2016, 2, 59–63.

[14] A. K. Soares , D. A. Gatto , W. L. E. Magalhães , X. Erdocia , M. A. U. Gutiérrez , R. A. Delucis , A. L. Missio , J. Wood Chem. Technol. 2021, 41, 199–209.

[15] J. Ruwoldt , Surfaces 2020, 3, 622–648.

[16] J. Ruwoldt , J. Planque , G. Øye , ACS Omega 2020, 5, 15007–15015. 32637774 10.1021/acsomega.0c00616 PMC7330892

[17] J. Zhang , Y. Ge , L. Qin , W. Huang , Z. Li , J. Dispersion Sci. Technol. 2018, 39, 1140–1143.

[18] N. Ghavidel , P. Fatehi , Langmuir 2021, 37, 3346–3358. 33667093 10.1021/acs.langmuir.0c03458

[19] Z. Zhang , Y. Zhang , Z. Lin , A. Mulyadi , W. Mu , Y. Deng , Chem. Eng. Sci. 2017, 165, 55–64.

[20] J. Ou , Z. Kong , R. Yang , Z. Dai , J. Dispersion Sci. Technol. 2021, 1–8.

[21] C. Gupta , N. R. Washburn , Langmuir 2014, 30, 9303–9312. 25046477 10.1021/la501696y

[22] N. Ghavidel , P. Fatehi , ChemSusChem 2020, 13, 4567–4578. 32419354 10.1002/cssc.202000965

[23] C. Shi , S. Zhang , W. Wang , R. J. Linhardt , A. J. Ragauskas , ACS Sustainable Chem. Eng. 2020, 8, 22–28.

[24] J. Yu , L. Li , Y. Qian , H. Lou , D. Yang , X. Qiu , Ind. Eng. Chem. Res. 2018, 57, 15740–15748.

[25] Y. Wu , Y. Qian , H. Lou , D. Yang , X. Qiu , ACS Sustainable Chem. Eng. 2019, 7, 15966–15973.

[26] S. Gharehkhani , N. Ghavidel , P. Fatehi , ACS Sustainable Chem. Eng. 2019, 7, 2370–2379.

[27] L. Shuai , M. T. Amiri , Y. M. Questell-Santiago , F. Héroguel , Y. Li , H. Kim , R. Meilan , C. Chapple , J. Ralph , J. S. Luterbacher , Science 2016, 354, 329–333. 27846566 10.1126/science.aaf7810

[28] W. Lan , M. T. Amiri , C. M. Hunston , J. S. Luterbacher , Angew. Chem. Int. Ed. 2018, 57, 1356–1360; 10.1002/anie.201710838 29210487

[29] Y. M. Questell-Santiago , R. Zambrano-Varela , M. Talebi Amiri , J. S. Luterbacher , Nat. Chem. 2018, 10, 1222–1228. 30224685 10.1038/s41557-018-0134-4

[30] S. Bertella , J. S. Luterbacher , Green Chem. 2021, 23, 3459–3467.

[31] A. I. Benítez-Mateos , S. Bertella , J. Behaghel de Bueren , J. S. Luterbacher , F. Paradisi , ChemSusChem 2021, 14, 3198–3207. 34111325 10.1002/cssc.202100926 PMC8457236

[32] M. Talebi Amiri , G. R. Dick , Y. M. Questell-Santiago , J. S. Luterbacher , Nat. Protoc. 2019, 14, 921–954. 30778206 10.1038/s41596-018-0121-7

[33] D. S. Zijlstra , C. W. Lahive , C. A. Analbers , M. B. Figueirêdo , Z. Wang , C. S. Lancefield , P. J. Deuss , ACS Sustainable Chem. Eng. 2020, 8, 5119–5131.

[34] X. Meng , C. Crestini , H. Ben , N. Hao , Y. Pu , A. J. Ragauskas , D. S. Argyropoulos , Nat. Protoc. 2019, 14, 2627–2647. 31391578 10.1038/s41596-019-0191-1

[35] N. Delgado , F. Ysambertt , G. Chávez , B. Bravo , D. E. García , J. Santos , Waste Biomass Valorization 2019, 10, 3383–3395.

[36] S. Chen , Y. Zhou , H. Liu , J. Yang , Y. Wei , J. Zhang , Energy Fuels 2019, 33, 6247–6257.

[37] J. R. D. Montgomery , C. S. Lancefield , D. M. Miles-Barrett , K. Ackermann , B. E. Bode , N. J. Westwood , T. Lebl , ACS Omega 2017, 2, 8466–8474. 31457383 10.1021/acsomega.7b01287 PMC6645228

[38] A. Moreno , M. Morsali , J. Liu , M. H. Sipponen , Green Chem. 2021, 23, 3001–3014.

[39] X. Ouyang , L. Ke , X. Qiu , Y. Guo , Y. Pang , J. Dispersion Sci. Technol. 2009, 30, 1–6.

[40] N. Migliore , D. S. Zijlstra , T. G. Van Kooten , P. J. Deuss , P. Raffa , ACS Appl. Polym. Mater. 2020, 2, 5705–5715.

[41] Z. Zhao , W. Wang , J. Xiao , Y. Chen , Y. Cao , Nanomaterials 2020, 10, 1068. 10.3390/nano10061068 PMC7352959 32486322

[42] A. N. Evdokimov , A. V. Kurzin , O. V. Fedorova , P. V. Lukanin , V. G. Kazakov , A. D. Trifonova , Wood Sci. Technol. 2018, 52, 1165–1174.

[43] E. Schuler , M. Demetriou , N. R. Shiju , G.-J. M. Gruter , ChemSusChem 2021, 14, 3636–3664. 34324259 10.1002/cssc.202101272 PMC8519076

[44] K. Isobe , Biosci. Biotechnol. Biochem. 1995, 59, 576–581. 7772820 10.1271/bbb.59.576

[45] L. Manker, G. Dick, A. Demongeot, M. Hédou, C. Rayroud, T. Rambert, M. Jones, I. Sulaeva, Y. Leterrier, A. Potthast, F. Maréchal, V. Michaud, H.-A. Klok, J. Luterbacher, *Nat. Chem*. **2022** in press. DOI 10.1038/s41557-022-00974-5. 10.1038/s41557-022-00974-5 35739426

[46] X. Meng , C. Crestini , H. Ben , N. Hao , Y. Pu , A. J. Ragauskas , D. S. Argyropoulos , Nat. Protoc. 2019, 14, 2627–2647. 31391578 10.1038/s41596-019-0191-1

