# Supplementary material for: Extraction and Surfactant Properties of Glyoxylic Acid‐Functionalized Lignin
Source: ChemSusChem. 2022 Jun 7;15(15):e202200270. doi: 10.1002/cssc.202200270 (PMC9543430; doi:10.1002/cssc.202200270)
Supplement: Supplementary file 1 — Supporting Information [file CSSC-15-0-s001.pdf]

ChemSusChem

Supporting Information

Extraction and Surfactant Properties of Glyoxylic Acid-Functionalized Lignin

Stefania Bertella, Monique Bernardes Figueirêdo, Gaia De Angelis, Malcolm Mourez, Claire Bourmaud, Esther Amstad, and Jeremy S. Luterbacher*© 2022 The Authors. ChemSusChem published by Wiley-VCH GmbH. This is an open access article under the terms of the Creative Commons Attribution License, which permits use, distribution and reproduction in any medium, provided the original work is properly cited.

Table of Content

S1 Chemicals and materials

S2 Experimental Methods

- S2.1 Biomass preparation
- S2.2 Calculation of Effective Extracted lignin
- S2.3 Propionaldehyde (PA)-lignin extraction
- S2.4 Characterization of lignin samples
  - S2.4.1 Quantitative ^{31}P-NMR
  - S2.4.2 Diffusion-ordered spectroscopy (DOSY-NMR)
  - S2.4.3 Impurities quantification by High-Pressure Liquid Chromatography (HPLC)
- S2.5 Dynamic Light Scattering (DLS) measurements
- S2.6 Microscopy imaging of emulsions and photos of emulsion vials over time
- S2.7 Surface Tensions of different lignin and fossil-based surfactants
- S2.8 UV-vis measurements of lignin solutions at pH 4

MATERIALS AND METHODS

S1 Chemicals and materials

All commercial chemicals were of analytical grade and were used without further purification. 2-Chloro-4,4,5,5-tetramethyl-1,3,2-dioxaphospholane 95% (TMDP), Mineral Oil, sodium hydroxide, sodium bicarbonate, Sudan Black B certified by Biological Stain Commission were purchased from Sigma Aldrich. 1,4-Dioxane, Sodium lignosulfonate 93% (pulverized) and Calcium lignosulfonate 93% (pulverized) were purchased from Carl Roth. Chromium (III) acetylacetonate 97%, Pyridine 99.5%, Glyoxylic acid monohydrate 98% (GA), propionaldehyde 99+% (PA), and Cyclohexane 99.5% were purchased from Acros Organics. Chloroform-d_3 99,8% ($CDCl_3$) and Dimethylsulfoxide-d_6 (DMSO-d_6) were purchased from Cambridge Isotope Laboratories. Hydrochloric acid 37% w/w (HCl) and tetrahydrofuran (THF) stabilized with 0.025% w/w of BHT were purchased from Fisher Chemical. The Kraft Lignin in this work was UPM's BioPiva™ 100, which was dried in a vacuum oven at 45°C for 24h before its use. Birch (*Betula Pendula*) wood chips were supplied by Prof. Michael Studer from the Bern University of Applied Sciences. The tree was harvested in May 2018 in Solothurn, Switzerland. Beech (*Fagus sylvatica*) wood was provided by a Swiss woodcutter, Jimmy Rochat.

S2 Experimental Methods

S2.1 Biomass preparation

The Birch tree trunk was debarked, cut into wood chips and dried at 40°C for 24h. Afterwards, the wood chips were transported to EPFL (Lausanne, Switzerland) where they were separated from residual leaves and bark, milled with a 6mm screen and sieved with a 0.45 mm mesh. The Beech tree trunk was debarked and then chipped to form particles approximately 5 cm wide. These wood chips were then spread on a tarp for two weeks to dry before being reduced using a Retsch cutting mill (6mm grid).

S2.2 Calculation of Effective Extracted lignin

The yields of effective extracted lignin shown in **Table S1** are calculated based on the composition of the same birch wood used in the work previously published by Talebi Amiri et al.[1] and according to the following equations:

$$\textbf{Effective isolated lignin} = \mathrm{g_{isolated\ lignin}} - \mathrm{n_{bound\ GA}} \times 58.01\mathrm{g/mol} \qquad \text{(Equation S1)}$$

$$\textbf{Lignin Extraction Yield}_{\boldsymbol{Klason}}\ [\textbf{wt.}\%] = \frac{\text{Effective isolated lignin}}{\text{Original Klason Lignin Content}}\mathrm{x}100 \qquad \text{(Equation S2)}$$

Where in the equations:

$g_{isolated\ lignin}$: mass of isolated lignin

$n_{bound\ GA}$: mmol of GA covalently bound to the isolated lignin (measured by ^{31}P NMR according to **section S2.5.2**)

The value 58.01 g mol^{-1} corresponds to the molecular weight of a GA molecule when covalently bound to the lignin.

Table S1. Experimental data and corresponding yields for the GA-lignin samples

Sample	Dry biomass in reactor [g]	GA monohydrate in reactor [g]	GA/dry biomass [mmol g^{-1}]	Lignin extraction yield (on Klason Lignin basis) [%]
**1**	4.699	0.372	0.859	78%
**2**	4.709	0.752	1.735	55%
**3**	4.715	1.356	3.124	65%
**4**	4.701	1.818	4.200	77%
**5**	4.731	2.773	6.366	71%
**6**	4.781	4.179	9.497	77%
**7**	4.717	5.073	11.684	61%
**8**	9.391	11.001	12.725	74%

S2.3 Propionaldehyde (PA)-lignin extraction

The extraction of PA-lignin was performed following a procedure published by Lan et al.[2] Briefly, 20g of beech wood chips were added into a 500 mL round bottomed flask along with 180 mL of dioxane, 19 mL of propionaldehyde (15.2 g, 261 mmol), 8.4 mL of HCl 37 wt.% (100 mmol) and a magnetic stirrer. The reaction mixture was heated to 85°C in an oil bath and stirred for 3h. Afterwards, the solution was cooled to room temperature and neutralized with 8.4g of $NaHCO_3$ (100 mmol). The reaction mixture was left to stir for an additional 30 minutes and was then filtered on a Buchner to separate the insoluble cellulose-rich fraction from the soluble lignin and hemicellulose fractions. The filtrate was concentrated with a rotary evaporator at 45°C and 50 mbar for approximately 20 minutes until its viscosity had noticeably increased. This mixture was then precipitated dropwise in 200 mL of diethyl ether to yield the propionaldehyde-

stabilized lignin. The lignin was finally filtered on a Buchner, washed with fresh diethyl ether, and dried at 45°C in a vacuum oven overnight.

S2.4 Characterization of lignin samples

A HSQC spectrum of Propionaldehyde functionalized lignin (PA-lignin) is shown in **Figure S1**.

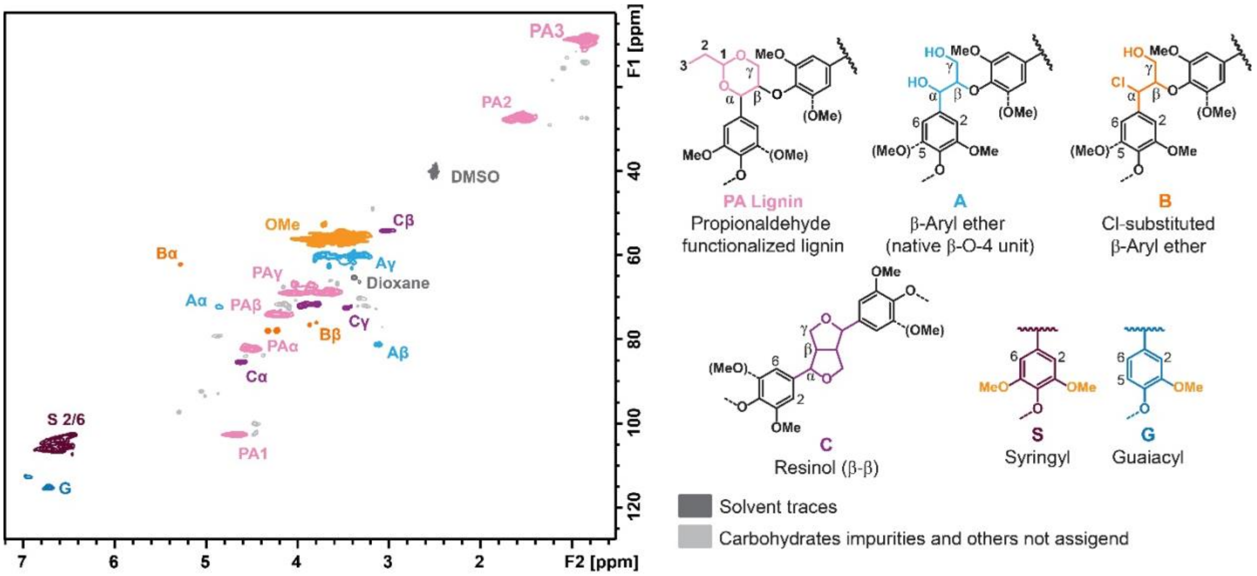

Figure S1. HSQC spectrum of propionaldehyde functionalized Lignin (PA-lignin)

S2.4.1 Quantitative ^{31}P-NMR

The quantified values of hydroxyl groups in the extracted GA-lignin samples via ^{31}P NMR are shown in **Table S2**.

Table S2. Quantification of aliphatic, phenolic and carboxylic hydroxyl groups of extracted GA-lignins via ^{31}P NMR. *Aliph-OH* refers to the aliphatic hydroxyl groups, *5-Subst.-OH* refers to phenolic groups on aromatic rings substituted at the position 5 (for example Syringol units), *G-OH* refers to phenolic groups of Guaiacol units, *H-OH* refers to phenolic groups of Hydroxyphenylpropane units and *COOH* refers to carboxylic acid groups.

Sample	Aliph-OH [mmol g^{-1}]	5-Subst.-OH [mmol g^{-1}]	G-OH [mmol g^{-1}]	H-OH [mmol g^{-1}]	COOH [mmol g^{-1}]
**1**	3.11	0.97	0.41	0.18	0.37

**2**	3.62	0.71	0.38	0.12	0.46
**3**	3.04	0.67	0.37	0.11	0.55
**4**	2.84	0.80	0.36	0.10	0.68
**5**	2.34	0.71	0.37	0.09	0.89
**6**	2.50	0.69	0.33	0.06	0.88
**7**	2.63	0.51	0.29	0.07	0.84
**8**	1.78	0.31	0.20	0.01	0.86
**PA-lignin**	1.72	0.70	0.29	0.00	0.13

S2.4.2 Diffusion-ordered spectroscopy (DOSY-NMR)

To assess the stability of the extracted lignin in water at pH 14, a DOSY (Diffusion Ordered SpectroscopY) pulse sequence (Bruker, ledbpgp2s) was run in NaOD 1M, D2O on a Bruker Avance-400, equipped with a BBI probe. Considering the high concentration of salts, the shim was performed manually and 90°C pulses (p1) carefully calibrated: 20 µsec on day 1, 19.1 µsec on day 14 and 15.1 µsec for guaiacol.

The DOSY parameters, i.e. the diffusion time interval (d20) and gradient pulse length (p30), were set at 0.1 s and 2000 ms, respectively, after optimization using the 1D DOSY ledbpgp2s1d pulse sequence. A relaxation delay (D1) of 3 sec was selected, allowing for complete relaxation between scans. Each 1D spectra was processed from a free induction decay with 16K complex points and 16 scans averaged. The diffusion gradient pulses were incremented from 2% to 98% using a linear ramp to generate 32 increments. All experiments were performed at a temperature of 298K. Finally, diffusion coefficients were calculated from the log of y axis (log (D), where D is the diffusion coefficient in m^2 s^{-1}). The NMR tube containing the sample was stored at room temperature on a lab bench between the two DOSY measurements.

For the calculation of D, we decided to focus on region of the aromatic signals of lignin and particularly at 6.5 ppm, as signals in the aliphatic region could be affected by the presence of residual carbohydrates as well as the solvent peak. The spectra obtained are shown in **Figure S2**.

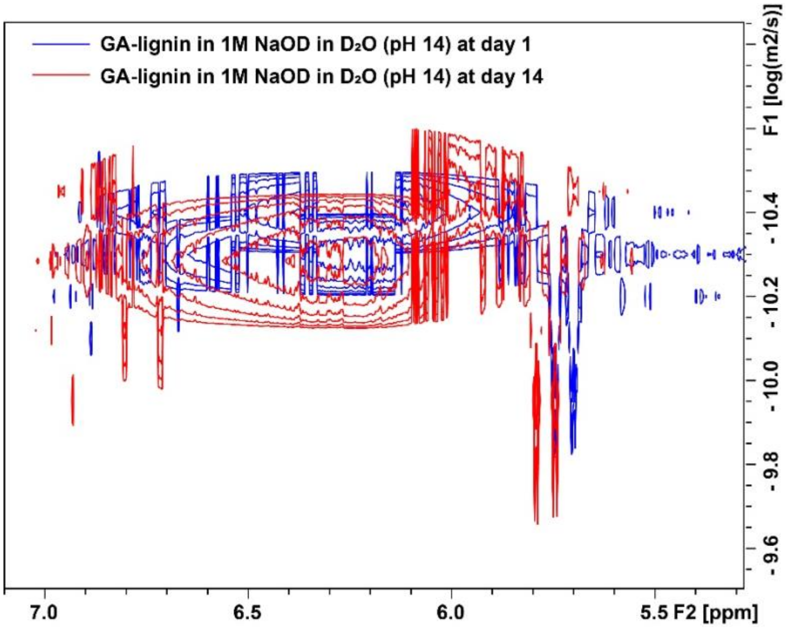

Figure S2. The DOSY spectra of GA-lignin in 1M NaOD in D_2O at pH 14 at day 1 (blue) and day 14 (red) in the region between 5.5 and 7 ppm.

From the spectra obtained we could calculate the D of GA-lignin at pH 14 at day 1 and 14. Comparing the values with the D calculated from the DOSY spectrum of Guaiacol (as a model compound for a lignin monomer) in the same solvent system. The results are shown in **Table S3**.

Table S3. Values of D calculated for GA-lignin in 1M NaOD in D_2O at pH 14 at day 1 and day 14 at 6.5 ppm, as well as the D of guaiacol in the same solvent system.

Sample	ppm	D [m^2 s^{-1}] Day 1	D [m^2 s^{-1}] Day 14
Ga-lignin at pH 14	6.5	4.71E-11	5.19E-11
Guaiacol	6.5	4.80E-10	n.d.

S2.4.3 Impurities quantification by High-Pressure Liquid Chromatography (HPLC)

To quantify residual impurities in GA-lignin (sample 8) we suspended 30 mg of lignin in 3 mL of an aqueous solution containing HCl 37 wt.% at pH 2. After vigorous stirring and sonication for 10 minutes, the lignin suspension was filtered through a syringe equipped with a PTFE syringe filter with a pore size of 0.22 µ. The liquid phase was then analyzed by HPLC using an Agilent Infinity 1260 HPLC system equipped with a Refractive Index (RI) Detector, a UV-Vis Detector and a Bio-Rad Aminex HPX-87H column. The

analysis was performed at 60°C using 5 mM H2SO4 in water as the mobile phase, and a flow rate of 0.6 mL min^{-1}.

The obtained chromatogram (**Figure S3**) presented three peaks. Two of these peaks at 9.889 min and 10.370 min were attributed respectively to Di-Glyoxylic Acid protected Xylose (recently reported by Manker et al.[3]) and GA. A third peak was not identified but we hypothesize being a derivative of a reaction between GA and other carbohydrates present during biomass fractionation.

We quantified the identified compounds by performing calibration curves. The results are presented in **Table S4**.

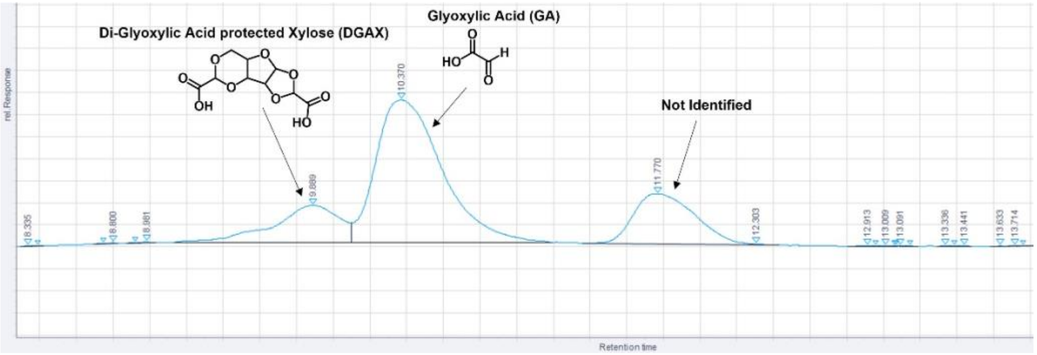

Figure S3. The HPLC chromatogram of the analysed GA-lignin.

Table S4. Assigned peaks and their quantification via HPLC.

Retention Time (min)	Compound assigned	Concentration (g g^{-1} of GA-lignin)
9.889	Di-Glyoxylic Acid protected Xylose	0.0009
10.370	Glyoxylic Acid	0.004
11.770	Not Identified	n.a.

S2.5 Dynamic Light Scattering (DLS) measurements

DLS measurements were made with a Zetasizer Nano-ZS apparatus (Malvern, UK). The analysed solutions were loaded into a plastic UV/visible absorbance spectroscopy cuvette and inserted into the machine. The data was then processed using the Zetasizer software provided by Malvern (v. 7.02). All solutions had an attenuation factor in the acceptable 6-9 range. Aqueous solutions/colloids of GA-lignin at pH 1, 7 and 14 were analyzed by DLS (**Figure S4**).

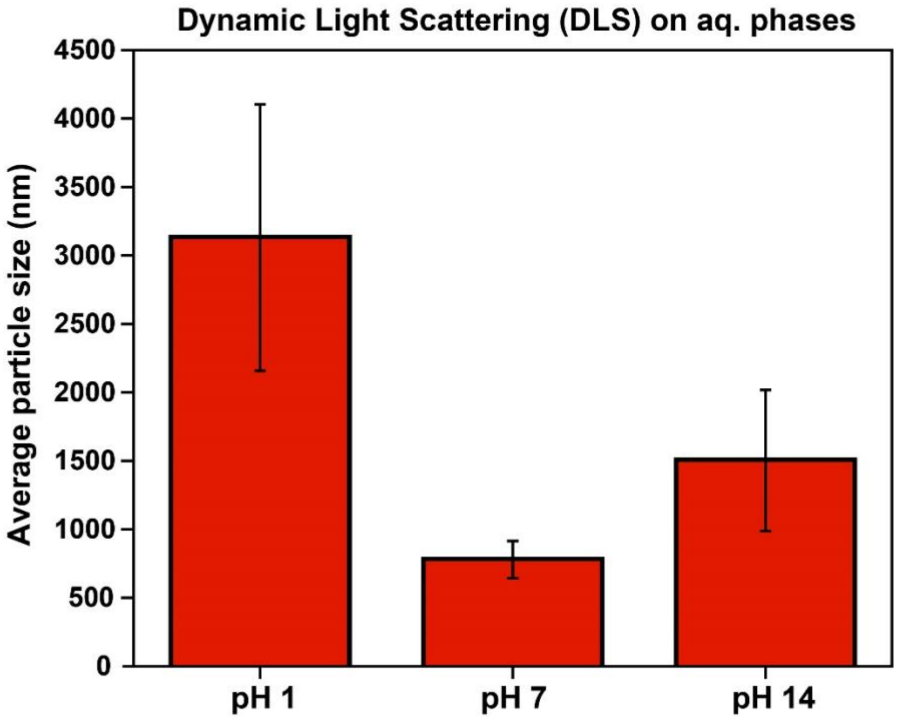

Figure S4. DLS measurements of lignin in aqueous phases at different pH values.

S2.6 Microscopy imaging of emulsions and photos of emulsion vials over time

Fluorescence images of the emulsion were taken with the same instrument and an example is shown in **Figure S5.**

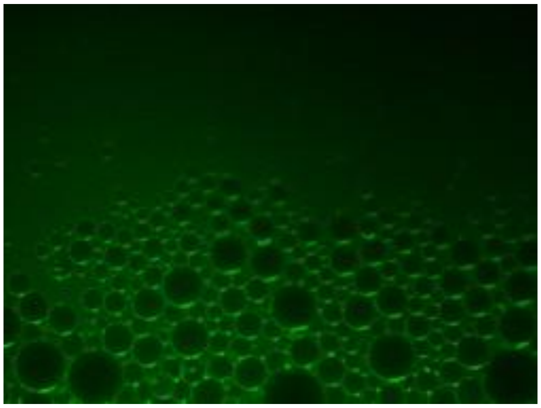

Figure S5. Fluorescence microscopy image of a cyclohexane/water emulsion. No fluorescence is observed inside the oil droplets confirming that this is an oil in water emulsion (as the autofluorescent lignin is in the water phase, not in the oil phase).

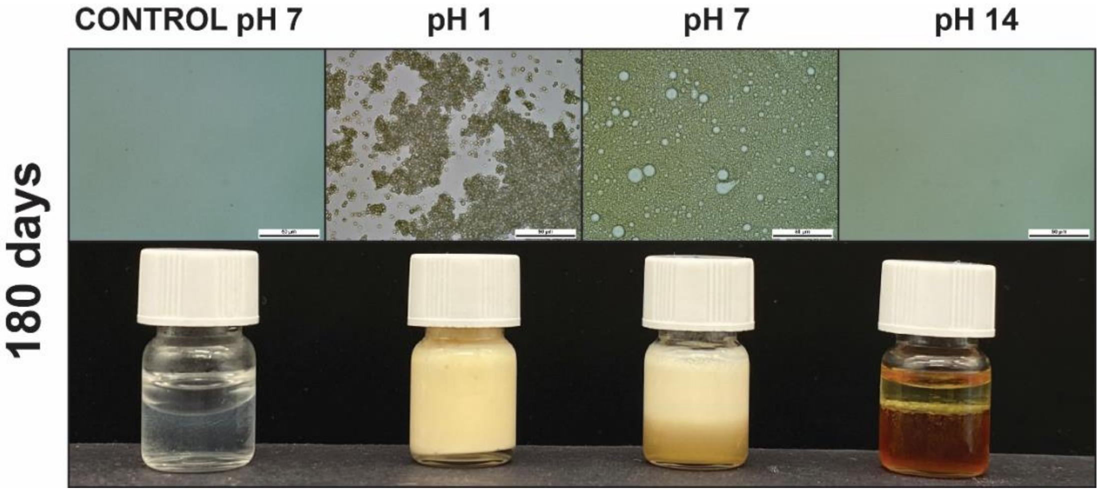

Figure S6. Images of water/mineral oil emulsions at different pH taken after 180 days: (Top) taken with optical microscopy with a scale bar of 50 µm. (Bottom) taken with traditional photography of the full vials containing the emulsions.

S2.7 Surface Tensions of different lignin and fossil-based surfactants

Table S5. Surface Tensions comparison of different lignins and industrial fossil-based surfactants

Sample	Surface Tension [mN/m]
GA-Lignin pH 14	32.3
GA-Lignin pH 7	31
GA-Lignin pH 1	52.7
Dodecyl Succinic Acid Grafted Lignin[4]	36
Kraft Lignin	37.4
Polycrylamide-grafted Butanosolv Lignin[5]	45
PEG-grafted Enzymatic Lignin[6]	50
Sulfomethylated Kraft Lignin[7]	51
Sodium Lignosulfonate	53.5
Sodium Dioctyl Sulfosuccinate	25.1
Sodium Dodecylbenzene Sulfonate	30.1
Sodium Dodecylsulfate	35.4

S2.8 UV-vis measurements of lignin solutions at pH 4

The UV-Vis spectra of Kraft Lignin and GA-lignin in aqueous solutions at pH 4 were measured by using a VWR UV-3100PV Spectrophotometer and quartz cuvettes. The lignin samples were prepared at a concentration of 0.03mg mL^{-1} to allow for an Absorbance value to not exceed 2.5. An aqueous solution of HCl at pH 4 was used as a blank control. The spectra were collected in the range of 200-900 nm with a scan every 0.5nm. The obtained spectra are shown in **Figure S7**.

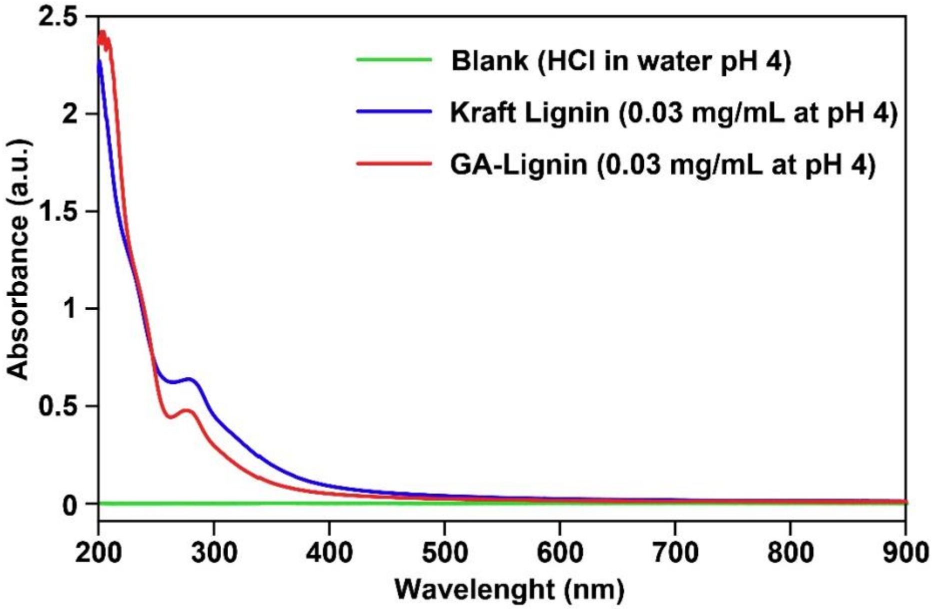

Figure S7. UV-Vis spectra of Kraft Lignin (blue) and GA-lignin (red) in aqueous solutions at pH 4 using An aqueous solution of HCl at pH 4 was used as a blank control (green).

References

[1] M. Talebi Amiri, G. R. Dick, Y. M. Questell-Santiago, J. S. Luterbacher, *Nat. Prot.* **2019**, *14*, 921–954.
[2] W. Lan, M. T. Amiri, C. M. Hunston, J. S. Luterbacher, *Angew. Chem., Int. Ed.* **2018**, *57*, 1356–1360.
[3] L. Manker, G. Dick, A. Demongeot, M. Hédou, C. Rayroud, T. Rambert, M. Jones, I. Sulaeva, Y. Leterrier, A. Potthast, F. Maréchal, V. Michaud, H.-A. Klok and J. Luterbacher, *ChemRxiv*, **2021**. This content is a preprint and has not been peer-reviewed.
[4] N. Delgado, F. Ysambertt, G. Chávez, B. Bravo, D. E. García, J. Santos, *Waste Biomass Valorization* **2019**, *10*, 3383–3395.
[5] N. Migliore, D. S. Zijlstra, T. G. Van Kooten, P. J. Deuss, P. Raffa, *ACS Appl. Polym. Mater.* **2020**, *2*, 5705–5715.
[6] C. Shi, S. Zhang, W. Wang, R. J. Linhardt, A. J. Ragauskas, *ACS Sustainable Chem. Eng.* **2020**, *8*, 22–28.
[7] X. Ouyang, L. Ke, X. Qiu, Y. Guo, Y. Pang, *J. Dispersion Sci. Technol.* **2009**, *30*, 1–6.
